# Effects of cnidarian biofouling on salmon gill health and development of amoebic gill disease

**DOI:** 10.1371/journal.pone.0199842

**Published:** 2018-07-06

**Authors:** Nina Bloecher, Mark Powell, Sigurd Hytterød, Mona Gjessing, Jannicke Wiik-Nielsen, Saima N. Mohammad, Joachim Johansen, Haakon Hansen, Oliver Floerl, Anne-Gerd Gjevre

**Affiliations:** 1 SINTEF Ocean, Trondheim, Norway; 2 Norwegian Institute for Water Research, Bergen, Norway; 3 Norwegian Veterinary Institute, Oslo, Norway; Friedrich-Loeffler-Institut Bundesforschungsinstitut fur Tiergesundheit, GERMANY

## Abstract

This study examines the potential implications of biofouling management on the development of an infectious disease in Norwegian farmed salmon. The hydroid *Ectopleura larynx* frequently colonises cage nets at high densities (thousands of colonies per m^2^) and is released into the water during regular *in-situ* net cleaning. Contact with the hydroids’ nematocysts has the potential to cause irritation and pathological damage to salmon gills. Amoebic gill disease (AGD), caused by the amoeba *Paramoeba perurans*, is an increasingly international health challenge in Atlantic salmon farming. AGD often occurs concomitantly with other agents of gill disease. This study used laboratory challenge trials to: (1) characterise the gill pathology resulting from the exposure of salmon to hydroids, and (2) investigate if such exposure can predispose the fish to secondary infections–using *P*. *perurans* as an example. Salmon in tanks were exposed either to freshly ‘shredded’ hydroids resembling waste material from net cleaning, or to authentic concentrations of free-living *P*. *perurans*, or first to ‘shredded’ hydroids and then to *P*. *perurans*. Gill health (AGD gill scores, non-specific gill scores, lamellar thrombi, epithelial hyperplasia) was monitored over 5 weeks and compared to an untreated control group.

Nematocysts of *E*. *larynx* contained in cleaning waste remained active following high-pressure cleaning, resulting in higher non-specific gill scores in salmon up to 1 day after exposure to hydroids. Higher average numbers of gill lamellar thrombi occurred in fish up to 7 days after exposure to hydroids. However, gill lesions caused by hydroids did not affect the infection rates of *P*. *perurans* or the disease progression of AGD. This study discusses the negative impacts hydroids and current net cleaning practices can have on gill health and welfare of farmed salmon, highlights existing knowledge gaps and reiterates the need for alternative approaches to net cleaning.

## Introduction

Gill diseases are one of the major health challenges in Atlantic salmon (*Salmo salar*) farming worldwide and can cause significant production losses [1–4]. Gill diseases often have a multifactorial aetiology [3–5] and there are various infectious and non-infectious agents that can compromise gill health, including viruses, bacteria, parasites, phyto- and zooplankton species, and biofouling organisms attached to the cage nets [1, 2, 6].

Amoebic Gill Disease (AGD), caused by the amoeba *Paramoeba perurans* (syn. *Neoparamoeba perurans*), is a prevalent disease in farmed salmon in Australia. Over the past years it has also caused considerable concern in Scottish, Irish, Chilean and Norwegian aquaculture [7]. Following the first discovery of AGD and *P*. *perurans* in Norway in 2006 [8] neither the disease nor the amoeba were diagnosed during the following 5 years. However, since 2012, AGD has been a persistent issue on salmon farms located on the west coast of Norway. AGD presents as raised white mucoid patches on the gills overlaying multifocal hyperplastic plaques where the filament epithelium is hyperplastic, resulting in fusing of adjacent lamellae and a reduction of functional gill surface area [9]. AGD can be fatal to farmed salmon [7].

Other agents that have been associated with gill disease often occur concomitantly with AGD, for example infections with *Candidatus* Branchiomonas cysticola [10], *Ca*. Piscichlamydia salmonis [11], *Ca*. Clavichlamydia salmonicola, [12], *Ca*. Syngnamydia salmonis [13], *Desmozoon lepeophtherii* [14] and salmon gill pox virus (SGPV) [15]. Infestations of these agents in combination with *P*. *perurans* have been investigated only to a very limited extent [3], and their biological and economic impact is not fully understood. Other potential concomitantly occurring gill insults are due to non-infectious agents such as phytoplanktonic or biofouling organisms. Among the latter, biofouling cnidarians that grow on the cage nets pose a known health risk to farmed salmon [16]. Hydroids such as *Ectopleura larynx*, *E*. *crocea* (syn. *Tubularia* spp.) and others can be found in high abundance on nets of salmon farms worldwide, where they, together with other biofouling, reduce oxygen levels, cause deformation and volume reduction in nets, decrease cleaner fish effectiveness and impact fish welfare [17–19]. In Norway and most other global farming regions, regular *in-situ* net cleaning with high-pressure washers is therefore used to remove biofouling from cages nets [16, 20]. Biofouling organisms dislodged during cleaning operations are released into the sea as whole organisms or as fragments [21]. Depending on season and locality, net cleaning has to be performed 1-4 times per month [22, 23]. Farmers report that fish in cages that are being cleaned and–depending on the direction of the water currents–also in the neighbouring cages, show stressed behaviour (jumping, avoidance of the cleaner, loss of appetite), suggesting discomfort during cleaning.

A recent study identified that the exposure to polyps of *E*. *larynx* and fragments contained in net cleaning waste has the potential to cause irritation and damage to the gills of Atlantic salmon [24]. Unfortunately, the experiments were not fully conclusive due to a contaminated control treatment. However, the mechanism causing the damage is known: nematocysts, characteristic of the phylum *Cnidaria*, are pressurised cells that, upon contact, eject microscopic barbs and neurotoxins to immobilise and capture prey [25]. Helmholz et al. [26] showed that toxins extracted from nematocysts of jellyfish resulted in morphological change and death of cultured rainbow trout gill cells. Although it is established that nematocysts may compromise the gill epithelial barrier, it is not yet understood to what extent this may predispose affected tissues to secondary infections. In farmed sea bream, epithelial damage due to infestations with gill parasites are often followed by secondary microbial gill infections [27]. In farming locations where nematocyst-bearing biofouling species are abundant, and occurrences of gill diseases are frequent, it is particularly important to understand any such interactions.

This study used laboratory challenge trials to characterise in detail the gill pathology exposure to hydroids (*E*. *larynx*) causes in salmon, and further investigate if such exposure could predispose the fish to secondary infection with *P*. *perurans*.

## Materials and methods

### Ethics statement

The study was carried out in accordance with the EU Animal welfare act and the Norwegian Regulations on the use of animals in research. The experimental protocol was approved by the Norwegian Food and Safety Authority (Permit. No. 7259).

### Sourcing and maintenance of experimental salmon

Challenge trials were conducted at NIVA's Solbergstrand laboratory in Drøbak, Oslofjord from November 10^th^ to December 15^th^ 2015. Atlantic salmon smolt of commercial origin were acclimatised to full salinity (34 ppt) over the course of 4 weeks after being randomly distributed across eight laboratory tanks (500 L) with n = 50 fish per tank. Individual tanks were surrounded by a curtain of heavy plastic to ensure isolation of treatments. Tanks were supplied with UV-treated sea water pumped from 60 m depth from the Oslofjord at a flow rate of 450–500 Lh^-1^. The average water temperature in the tanks was 15°C; the average salinity was 34 ppt. Fish were hand fed twice daily, five days a week, at 1% of body weight per feed. A 12-hour photoperiod was maintained. During the experiment, the fish grew from an average length of 22 cm to 24 cm, and their weight increased from an average of 102 g to 142 g.

### Experimental design

**Fig 1:**
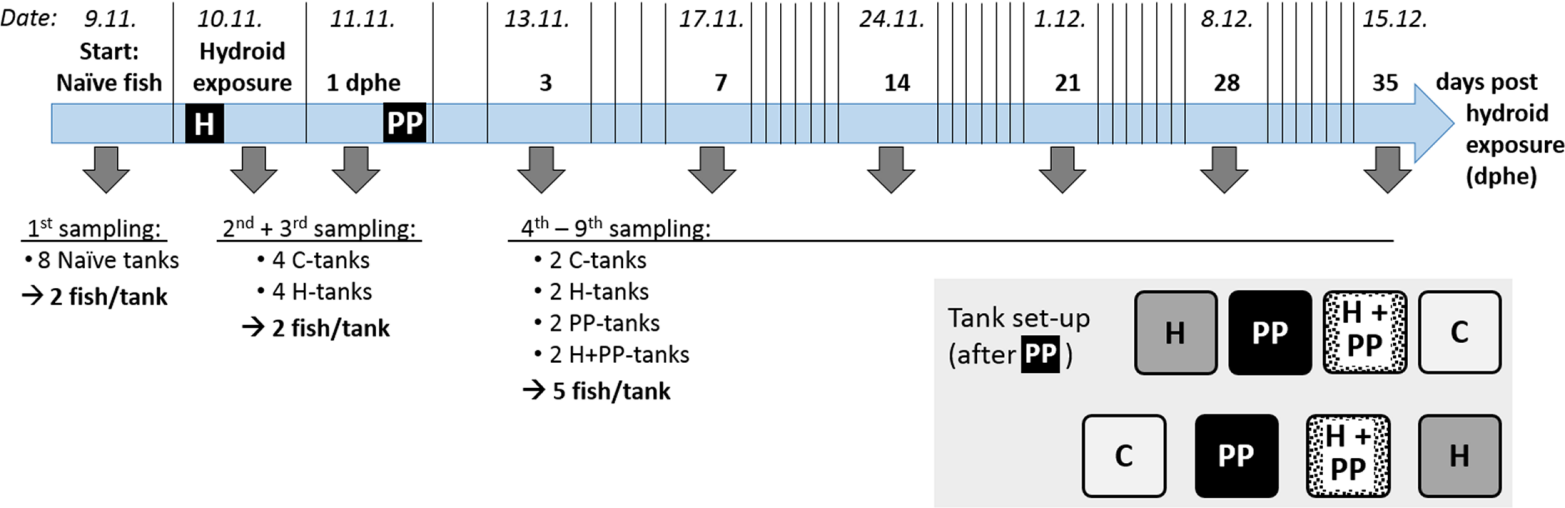
Experimental schedule showing sampling events (grey arrows) and numbers of sampled fish. Exposure to hydroids *Ectopleura larynx* ("H") took place one day post sampling of the naïve fish; exposure to *Paramoeba perurans* ("PP") took place one day post hydroid exposure (dphe). In addition, the distribution of the four treatment groups (C = Control, H = Hydroids, PP = *P*. *perurans*, H + PP = Hydroids + *P*. *perurans*) over the eight experimental tanks is shown. The numbers of sampled fish refer to each tank during each sampling event.

The challenge trial included four treatment groups, each of which was allocated two replicate tanks: (i) untreated salmon (control; hereafter referred to as ‘C-group’), (ii) salmon exposed to freshly ‘shredded’ hydroids resembling waste material from net cleaning (‘H-group’), (iii) salmon exposed to *P*. *perurans* (‘PP-group’), and (iv) salmon first exposed to ‘shredded’ hydroids and then to *P*. *perurans* (‘H+PP-group’; Fig 1). After exposure, gill health was monitored to establish the effect of hydroid exposure and whether exposure to hydroids affected *P*. *perurans* infection rates or disease progress of AGD in salmon.

### Hydroid growth and exposure

The aim was to create cleaning waste similar to that generated by *in-situ* high-pressure cleaning of fish cage nets. Based on field data on hydroid densities on fish cage nets ([21, 28]; SINTEF, unpublished data), combined with calculations of the water volume in an average sized fish farm cage (see S1 Appendix), a target concentration of 10 000 polyps per m^3^ was chosen for the present study to simulate conditions encountered on many Norwegian salmon farms. The hydroid *E*. *larynx* was cultivated on net panels (n = 12, 60x40 cm, uncoated nylon, 25 mm half mesh, Egersund Net) fixed to PVC frames (6 frames, 2 panels per frame) placed at 3 m depth at a salmon farm (Måsøval fiskeoppdrett) in Hemnfjorden (Mid-Norway), for 6 weeks. One day prior to the start of the experiment, 10 net panels were collected from the farm and placed into 25 L buckets filled with seawater (2 panels per bucket). The samples were aerated during car transport to Solbergstrand laboratory, as well as after transfer to fresh seawater upon arrival.

The density of *E*. *larynx* on the net panels was approximately 112 500 polyps m^-2^ (based on polyp counts on representative net strands conducted under a dissecting microscope; n = 10). The physical condition of the colony was good with most polyps in a reproductive state and only very few that had shed their hydranths. Other species, including bryozoans and small algae, contributed to less than 5% of the biofouling cover.

**Fig 2:**
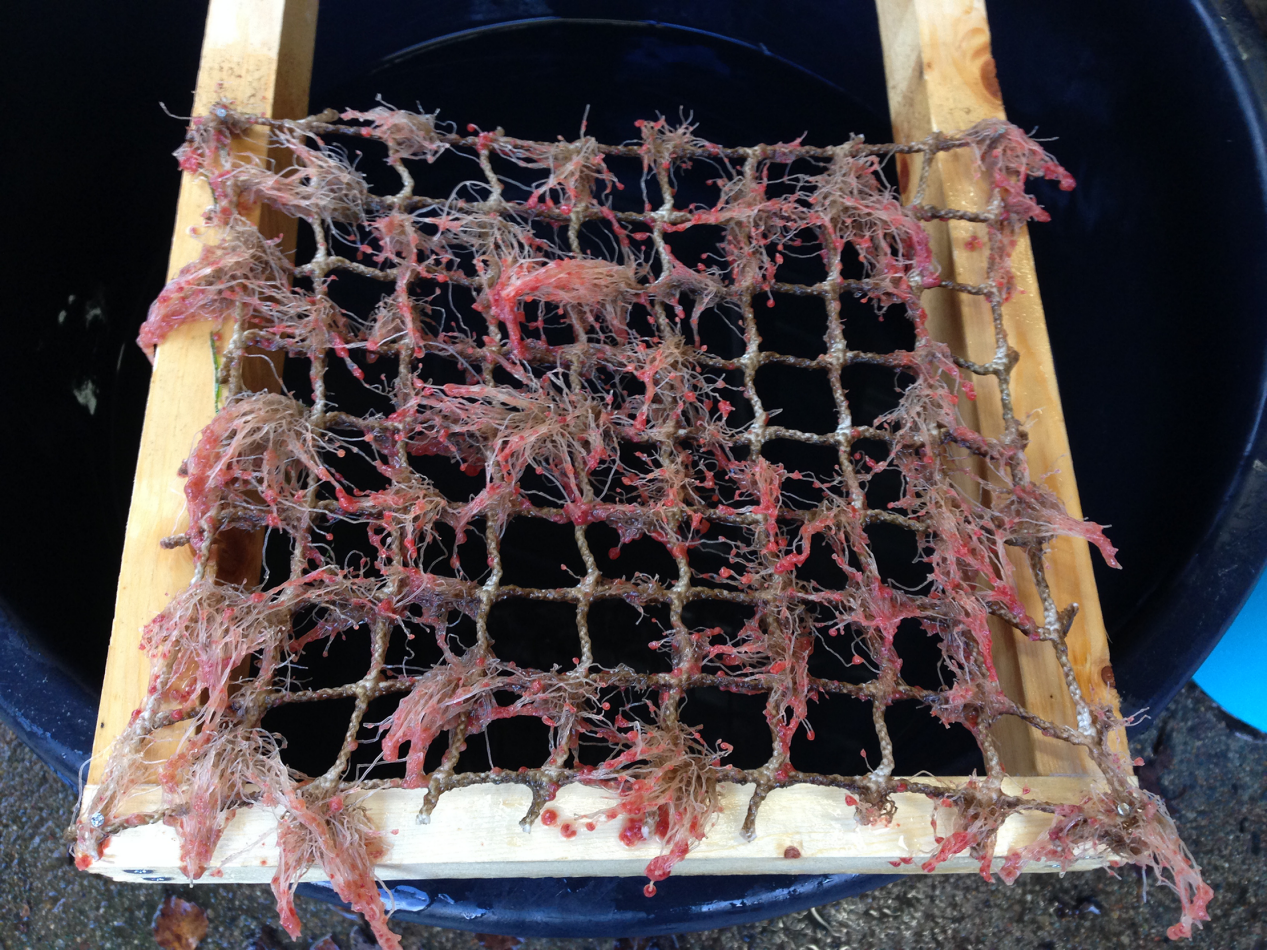
Hydroids on net sample attached to a frame for high-pressure cleaning.

Net panels with hydroids were mounted onto a wooden holding frame (Fig 2), which was then submerged into a large bucket with sea water. Biofouling was removed from the nets using a hand-held high-pressure cleaner supplying sea water with a pressure of 150 bar (Cocraft HHR 135, Clas Ohlson). The cleaner removed polyp heads and most stems effectively, but some remaining polyp stems had to be removed by hand. The cleaning waste was collected in the bucket and filtered through a 150 μm sieve to remove excess water. The approximate weight of the material in the sieve was recorded before the cleaning waste was divided into four equal parts and transferred to the experimental tanks designated for hydroid exposure.

Prior to exposure, the volume of water in each tank was set to 300 L and the water flow was turned off throughout the exposure period to ensure constant concentrations of cleaning waste. Pumps were used to generate water circulation in the tank to prevent settlement of hydroid material on the tank bottom. Cleaning waste exposure lasted for 3 hours, resembling an average *in-situ* net cleaning event (SINTEF; unpublished data). After the exposure period, the cleaning waste was removed from the tanks with nets and water flow-through was re-established.

Before, during, and after the exposure period, hydroid polyps (n = 5) were sampled to monitor the activity of the nematocysts. Polyps were mounted on microscope slides and 5% acetic acid was added to trigger nematocyst release [29]. In addition, entire hydroid polyps sampled before and after exposure to acetic acid were preserved in glutaraldehyde for scanning electron microscopy (SEM), which was performed using protocols described in Wiik-Nielsen et al. [30].

### Amoeba culture and exposure

*Paramoeba perurans* trophozoites were originally isolated from an infected Atlantic salmon gill arch according to Morrison et al. [31], with minor modifications. The isolated amoebae were maintained in cell culture flasks (Falcon 250 ml, canted neck, 75 cm^2^) in a malt-yeast broth (MYB: 0.01% malt extract, 0.01% yeast extract, sea water at 34 ppt salinity). To eliminate the possibility of including other amoeba species in the study, a monoclonal *P*. *perurans* culture was established according to the method of Crosbie et al. [32], seeding single amoeba cells to individual wells in a 96-well culture plate containing MYB. To obtain a sufficiently high density of amoebae for the exposure trials, the clonal isolate from one well was transferred to flasks and cultivated for 4 weeks prior to the experiment. The presence of *P*. *perurans* in the culture was confirmed by qPCR analyses using the assay of Downes et al. [33]. The amoeba challenge with *P*. *perurans* took place 1 day after exposure to hydroids (Fig 1). Fish were exposed to a concentration of 1500 amoebae L^-1^ for 1 hour. Prior to exposure, the waterflow was stopped and the level in all eight treatment tanks was reduced to 300 L during the exposure to ensure constant amoebae concentrations. One hour after addition of the amoebae, the water flow to the tanks was re-instated.

### Sampling of fish

Fish from all eight tanks were sampled haphazardly, using a dip net, (n = 2 fish per tank) once before (= naïve fish) and twice after hydroid exposure (1 hr and 24 hrs), but before exposure to *P*. *perurans* (Fig 1). Following the *P*. *perurans* challenge, fish were sampled six more times (n = 5 fish sampled per tank), with the last sampling 35 days post hydroid exposure (dphe) (Fig 1). Sampled fish were killed using an overdose of Metacaine at 0.1 g L^-1^ (Ethyl 3-aminobenzoate methanesulfonate, Sigma-Aldrich, Norway). Weight and length, gill score, skin lesions and sores were recorded. In addition, the following gill samples were collected: (i) filaments from the first gill arch on the left, preserved in RNA*later*® (QIAGEN, Hilden, DE), for qPCR analysis and (ii) second gill arch on the left, fixed in 10% neutral buffered formalin for histological analysis.

### Sample analysis

#### Gill scoring

Gills were scored macroscopically for non-specific injuries (incl. damage caused by nematocysts; Non-specific Gill Score, Table 1) and for AGD-related lesions (directly caused by amoeba; AGD Gill Score, Table 1), i.e. mucous patches or necrotic filaments, adapted from Taylor et al. [34]. Both sides of all eight gill arches were assessed and the highest score was noted.

**Table 1:** Descriptive and numeric scores corresponding to non-specific gill lesioning and AGD pathology, adapted from Taylor et al. [34].

Infection level	Gill Score Non-specific	Description	AGD Score	Description
**Clear**	0	No lesions visible	0	No sign of infection
**Very light**	1	Single necrotic filament or spot	1	1 white spot, light scarring or undefined necrotic streaking
**Light**	2	2–3 necrotic filaments	2	2–3 Spots/small mucus patches
**Moderate**	3	20 % of gill arch surface covered with necrotic tissue	3	Established thickened mucus patch or spot groupings up to 20% of the gill area
**Advanced**	4	20–50 % of the gill surface area covered with necrotic tissue	4	Established lesions covering up to 50 % of gill area
**Heavy**	5	> 50 % of the gill arch surface affected	5	> 50 % of gill area covered

#### Scoring of histopathological changes in the gills

For histological analysis gills were embedded in paraffin and a 3-μm thick section was cut and stained with haematoxylin and eosin (H&E). Additional staining with Martinus Scarlet and Blue (MSB) was used for verification of fibrin when the presence of lamellar thrombi was suspected based on the H&E stain. Each section was scored blinded, based on the degree (0 = absent, 1 = sparse, 2 = moderate, 3 = extensive) of lamellar thrombi (i.e., clotting of blood constituents, including fibrin, in the lamellar vessels), epithelial hyperplasia (i.e., excessive increase in the number of epithelial cells), specific AGD-related lesions (i.e., segmental epithelial hyperplasia and fusion of lamellae (with lacunae), [9]). In addition, the presence or absence of amoebae in the sample was noted.

#### qPCR

DNA was extracted from gill samples using the DNEasyKit (Qiagen^®^) on a QiaCube extraction robot (Qiagen^®^). To confirm the presence of *P*. *perurans*, gill samples of all fish were analysed using the qPCR assay as described by Downes et al. [33]. Gills of all naïve fish as well as the last two fish from each tank sampled at 21 and 35 dphe were also analysed for the presence of *'Candidatus'* Branchiomonas cysticola (Bacteria), *Desmozoon lepeophtherii* (Microsporidia), Salmon Gill Pox Virus (SGPV) using the qPCR assays in Nylund et al [35], Mitchell et al. [36], and Gjessing et al. [15], respectively. For all qPCR analyses, samples were considered positive if the Cq-values were below the cut-off-values provided in the respective publications.

#### Statistical analyses

Due to the difference in the numbers of fish sampled per tank, separate statistical analyses were performed for data (i) preceding and (ii) following exposure to *P*. *perurans*. The first analysis included the naïve fish and the two sampling events following exposure to hydroids ("Hydroid exposure" and "1 dphe", Fig 2). The second analysis included all six sampling events after exposure to *P*. *perurans* ("3 dphe" and onwards).

Differences in the magnitude of non-specific gill scores, AGD gill scores and the histological AGD lesion, thrombi and hyperplasia scores were analysed using permutational analysis of variance (PERMANOVA, PRIMER v.6.0). Similar to previous studies [6, 34], variance-based tests for the nominal scores were used since higher scores signify increased severity of disease. Analyses were based on Euclidian distance with 9999 unrestricted permutations of residuals under a reduced model. A significance level of 5% was used. Where the number of unique permutations was ≤ 100, the Monte-Carlo asymptotic p_MC_-value was consulted [37]. Since only two fish per tank were sampled proceeding the exposure to *P*. *perurans*, tanks could not be analysed individually and were therefore combined, resulting in a design with "Treatment" and "Time" as the two fixed factors. Following addition of *P*. *perurans* to the tanks, when 5 fish were sampled at each sampling date, "Tank" was included as a random factor. Where PERMANOVA indicated no significant differences between tanks (significance level of 25%) this term was pooled to increase power [37].

Fisher's Exact test was used to determine any relationships between the exposure of fish to hydroids and the prevalence of gill disorder or disease (gill, thrombi, and hyperplasia scores > 0). This test was also used to examine potential relationships between the prevalence of AGD-related gill damage (AGD scores > 0 and AGD lesions) and exposure to hydroids (i.e., comparisons between PP and H+PP groups). Average results are presented ± 1 Standard Error (SE). More detailed statistical results are presented in S2 Appendix; the original data can be found in S1 Table.

## Results

Over the course of the experiment mortality occurred in three of the 400 experimental salmon. Two of these mortalities occurred in control tanks (no hydroids or AGD exposure). The fish bore no obvious signs of disease or injury, suggesting the cause to be natural mortality.

### Nematocyst activity

**Fig 3:**
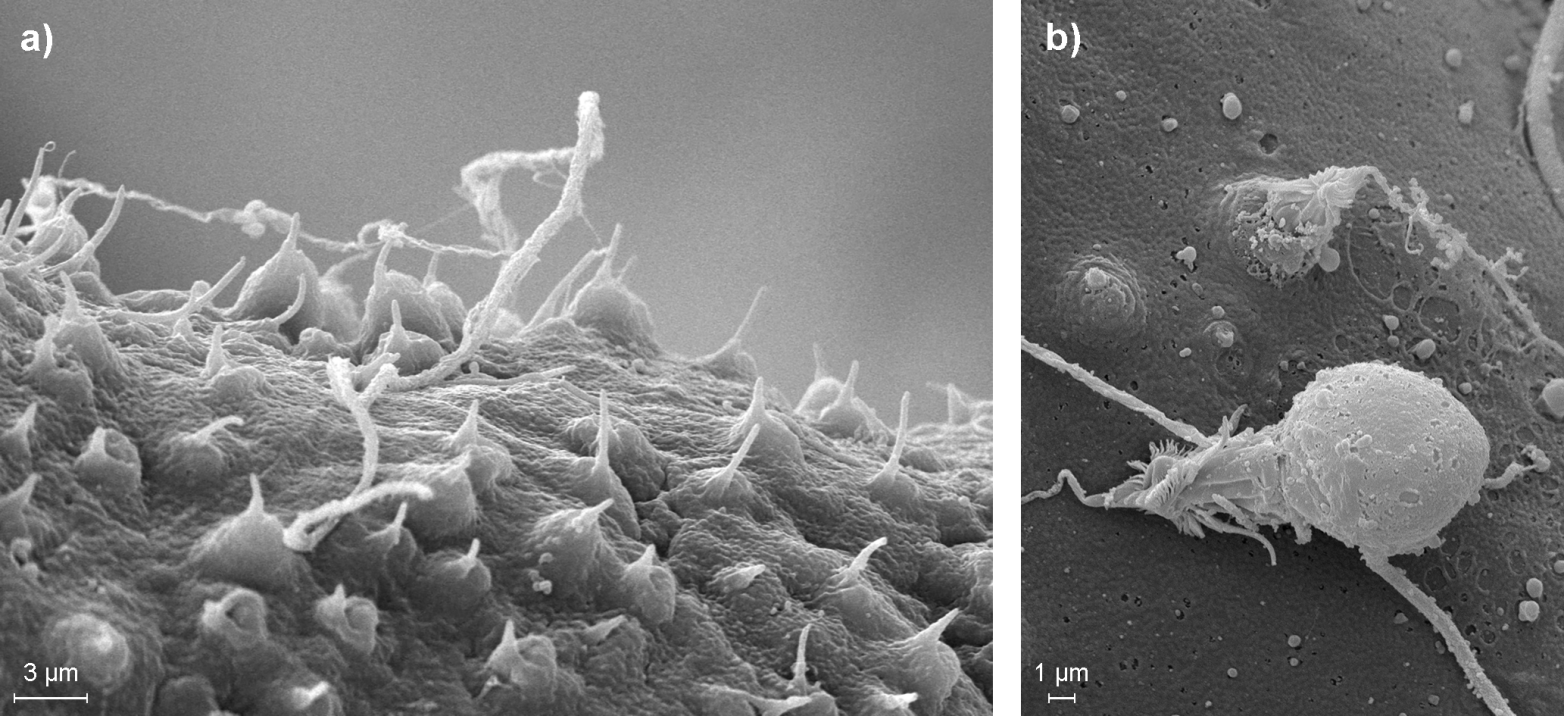
Scanning electron microscope images of a hydroid tentacle and nematocysts. a) Close-up of a tentacle of *E*. *larynx*, showing cnidocilia of undischarged nematocysts protruding the surface, ready to discharge on contact. b) Two discharged stenotele nematocysts (identified according to Östmann et al. [38]) found after triggering release with acetic acid.

All of the *Ectopleura larynx* polyps sampled before and after treatment with the high-pressure cleaner, as well as after the 3-hour exposure period in the tanks, successfully released nematocysts from their tentacles upon stimulation with acetic acid (Fig 3).

### Macroscopic gill lesions

#### Non-specific gill score

**Fig 4:**
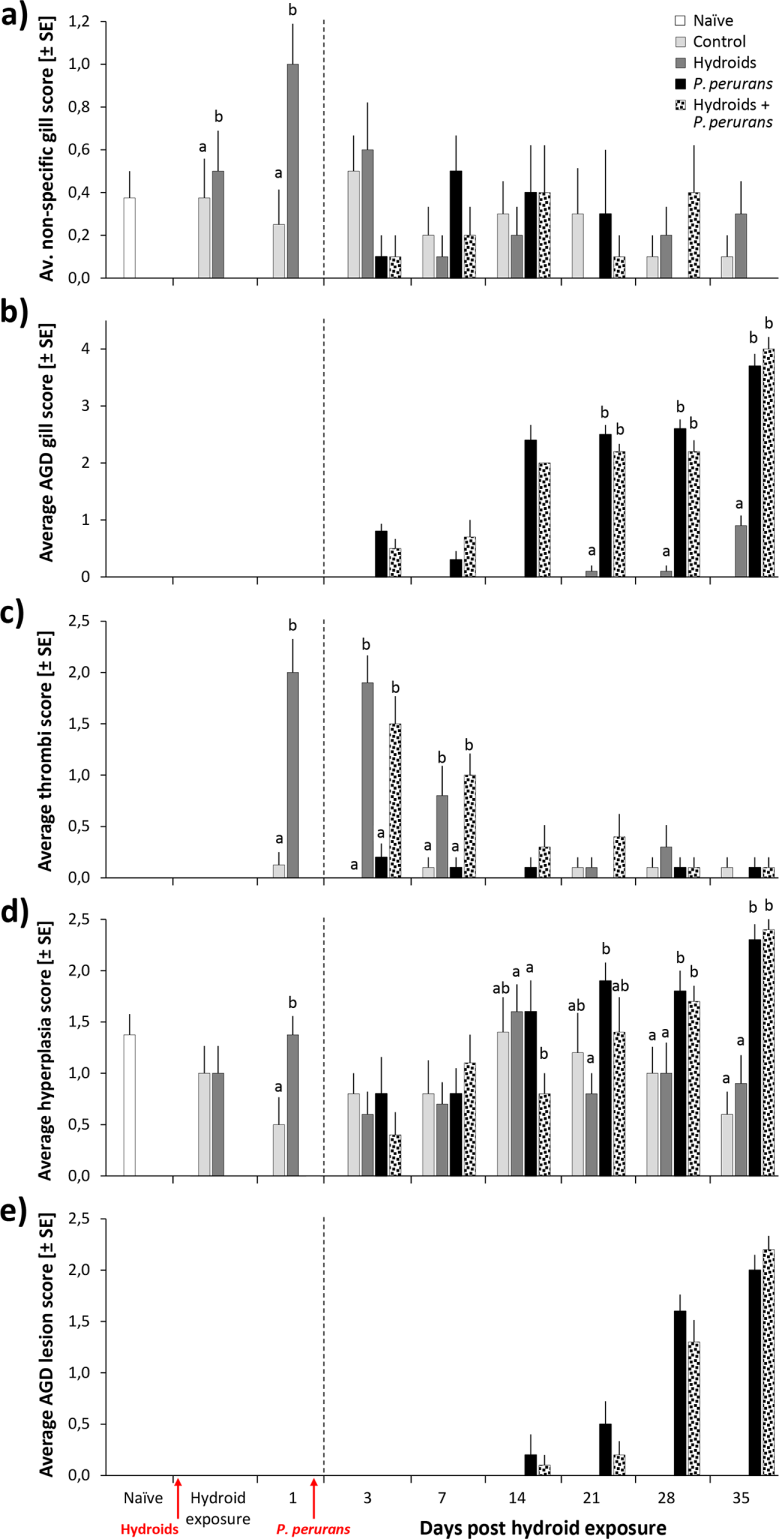
**Aspects of gill health, measured as (a) Non-specific gill score, (b) AGD gill score, (c) Thrombi score, (d) Hyperplasia score, (e) AGD lesion score.** All values are given as average ± SE. Timing of exposure to hydroids and *P*. *perurans* is indicated by arrows. Significant differences between treatments are indicated by letters representing results from (post hoc) comparisons. Left of the broken line two fish per tank were sampled; right of the broken line five fish were sampled.

Non-specific gill scores for macroscopic injury not related to AGD ranged from 0 to 3. At 1 dphe, the prevalence of fish with positive non-specific gill scores was significantly related to previous exposure to hydroid material, and considerably greater than in the control fish (C-group: 38%, H-group: 50%; p = 0.023). Also the average non-specific gill score was significantly higher for fish exposed to hydroids directly after exposure (0 dphe) and at 1 dphe (C-group: 0.4 ± 0.18 (SE) and 0.3 ± 0.16 vs. H-group: 0.5 ± 0.19 and 1.0 ± 0.19 at 0 and 1 dphe; respectively; F_1,28_ = 5.814; p = 0.025, Fig 4A). However, from 3 dphe onward, till the end of the experiment, there were no significant differences in average non-specific gill score between tanks or treatment groups (Fig 4A).

#### AGD gill score

AGD gill scores were zero in fish from the control group, and in all fish sampled prior to exposure to *P*. *perurans*. Post exposure to *P*. *perurans*, the AGD scores increased with time, with a maximum score of 5 being recorded.

At 3 dphe, the prevalence of AGD-scores > 0 was 80% in fish from the PP-group, and 50% in fish from the H+PP-group. From 14 dphe onwards, 100% of the fish in the PP and H+PP-group showed positive AGD scores. In the H-group (no *P*. *perurans* exposure), one fish with positive AGD score was found at 21 and 28 dphe. At 35 dphe, 80% of the fish sampled from both tanks of the H-group had positive AGD gill scores, indicating contamination of these tanks with *P*. *perurans*. The average AGD gill scores of fish from the PP and H+PP-group were significantly higher at all times than the average scores of fish from the contaminated H-group (Treatment x Time: F_15,192_ = 14.245; p < 0.001), but these differences were not always consistent between tanks (Tank(Treatment) x Time: F_15,192_ = 1.68; p = 0.04, Fig 4C). There were no differences in the AGD gill scores of fish from the PP and the H+PP-group.

qPCR analysis confirmed the presence of *P*. *perurans* in the PP and H+PP groups, as well as in the contaminated H-group.

### Scoring of histopathological changes in the gills

#### Gill lamellar thrombi

**Fig 5:**
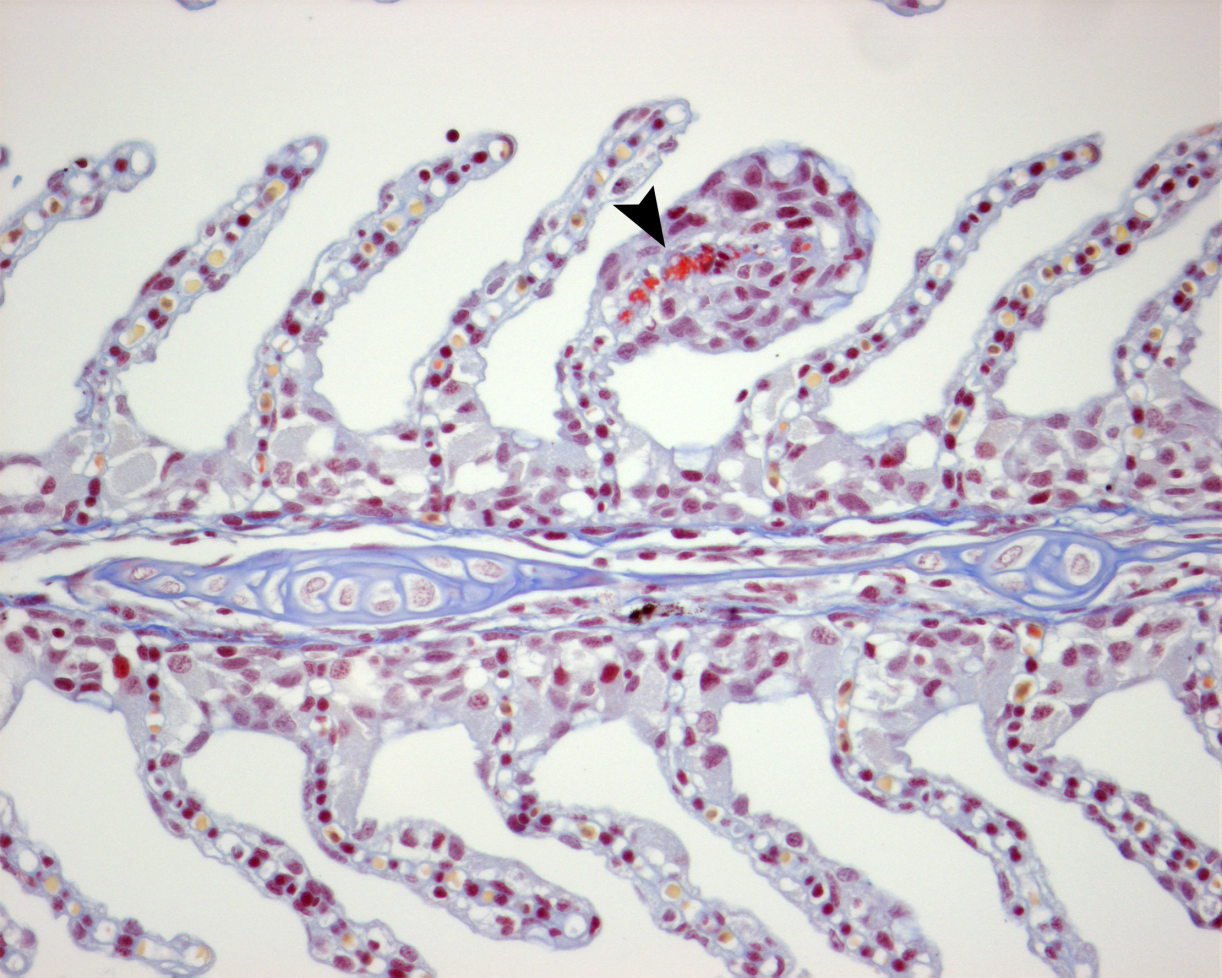
Gill filament of Atlantic salmon showing a lamellar thrombus after exposure to hydroids (H&E and MSB stained).

Lamellar thrombi (Fig 5) were seen in the gills from 1 dphe onwards; thrombi scores ranged from 0 to 3. At 1 dphe, the prevalence of lamellar thrombi (scores > 0) was significantly higher in the H-group (88%) than in the C-group (13%; p = 0.01). Also, average thrombi scores were higher for fish exposed to hydroids (H and H+PP groups, 0.8 to 2.0) than for fish without exposure (C and PP groups, 0 to 0.2) at 1 to 7 dphe (1 dphe: Treatment x Time: F_1,28_ = 28.64, p < 0.001; 3 & 7 dphe: Treatment x Time: F_15,216_ = 6.56; p < 0.001; pairwise post-hoc comparisons; Fig 4B). No differences in thrombi scores occurred between fish with or without exposure to *P*. *perurans* (C- vs. PP-group). After 7 dphe, the thrombi score declined in all four treatment groups to an average thrombi score below 1 and no differences between treatment groups occurred.

#### Gill epithelial hyperplasia

Fish showed a high prevalence of epithelial hyperplasia already before the start of the experiment (88% of naïve fish had scores > 0), with epithelial hyperplasia scores ranging from 0 and 3 throughout the experiment. Directly after exposure to hydroids (1 dphe), the prevalence of epithelial hyperplasia (scores > 0) increased to 100% (H-group), compared to 38% in the control group (C-group; p = 0.01). Average hyperplasia scores differed between treatment groups, but not at all sampling times (Treatment x Time: F_15,216_ = 2.94; p < 0.001). From 21 dphe onwards, fish from the PP-group, and from 28 dphe onwards also the fish from the H+PP-group, had significantly higher hyperplasia scores than the fish from the C and H groups (post-hoc pairwise comparisons, p < 0.05). Similar to the AGD gill scores, there was no difference in hyperplasia scores between fish from PP and H+PP groups (Fig 4d).

#### Histopathological changes consistent with AGD and presence of *P*. *perurans*

Histopathological changes consistent with AGD were detected only in fish from the PP and H+PP groups, and only from 14 dphe onwards. AGD lesion scores ranged from 0 to 3. The prevalence of AGD lesions (> 0) was not affected by exposure to hydroids (p > 0.05). No difference in average AGD lesion score occurred between fish from PP and H+PP groups (Treatment x Time: F_15,212_ = 28.46; p < 0.001; post-hoc pairwise comparisons of PP vs. H+PP; p < 0.05, Fig 4E).

Throughout all groups, background pathology consisted of some inflammatory and degenerative lesions.

*P*. *perurans* were identified only in the gill section of fish from the PP and H+PP groups. The earliest occurrence was at 14 dphe (2 fish), and by 28 dphe, 70–100% of the samples from the PP and H+PP groups showed the presence of *P*. *perurans*.

### Other gill pathogens

The qPCR assays returned negative results for *D*. *lepeophtherii* in all tested fish and confirmed the presence of *B*. *cysticola* in all tested fish. While the naïve fish were negative for SGPV, two fish tested positive at 21 dphe. At 35 dphe, SGPV was detected in all tanks of the four treatment groups, with 11 of 16 sampled fish testing positive.

## Discussion

This is the first study to examine the potential implications of biofouling management on the development of an infectious disease in farmed salmon. The study provides the first experimental evidence that nematocysts of the cnidarian *E*. *larynx* contained in cleaning waste and fragmented hydroid tissues can remain active after high-pressure treatment, and that exposure to such net cleaning waste can damage the gills of Atlantic salmon. However, pre-exposure to hydroids and the concomitant pathological changes to the gills did not affect the infection rates of *P*. *perurans* or the disease progression of AGD.

### Gill damage caused by hydroids

Contact of hydroid fragments with gill lamellae led to increased occurrence of thrombi in the gills up to 7 days after exposure. On a macroscopic level, injuries were visible for 24 hrs after exposure, with higher non-specific gill scores than in the control group. The most likely cause for the sustained damage observed in gills exposed to hydroid material are 'stings' caused by their nematocysts that cover the tentacles as well as the body of *E*. *larynx* in abundance (Fig 3), and that are released upon contact with potential prey or predator organisms [38, 39]. The presence of lamellar thrombosis seen in the fish of this study after exposure to hydroids correspond well to results from previous studies where fish have been exposed to nematocyst bearing jellyfish, both in the lab [6, 40, 41] and in the field [42–44]. Thrombi were not detected in the gills directly after exposure to *E*. *larynx*, but showed highest prevalence and severity 24 and 48 hours post-exposure, suggesting that these injuries take some time to manifest. Helmholz et al. [26] reported a similar increase in occurrence of morphological changes over the first 48 hours in rainbow trout gill cells after exposure to jellyfish nematocyst toxins. Similarly, Baxter et al. [44] recorded the highest gill scores 48 hours after exposure of salmon to *E*. *larynx*.

These histopathological patterns are likely caused by both the mechanical damage of the nematocysts penetrating the gill tissue, similar to spiky algal cells that cause irritation to gills [2], as well as the toxins released from the nematocyst, which have been shown to result in cell death when isolated and added to gill cell cultures [26].

An important finding of this study is that high pressure water treatment of hydroid colonies on nets did not trigger or incapacitate the hydroids’ nematocysts. Stinging cells on hydroid polyps and fragments remained active throughout, and until > 3h following the cleaning process. Thus, net cleaning using high pressure water jets, and the subsequent spread of suspended cleaning waste particles containing hydroids through production cages, comprises a health and welfare risk for cultured salmon. Exposure to jellyfish causes, among other symptoms, jumping behaviour and loss of appetite [2, 42]. Both reactions have been reported repeatedly by Norwegian salmon farmers during net cleaning in locations where hydroids were abundant (SINTEF, unpublished data), implying a reaction similar to that described for jellyfish. After exposure to *E*. *larynx*, gills showed significantly higher levels of thrombi for up to 7 days. With net cleaning being conducted sometimes as often as weekly during the main biofouling season [16], farmed fish may in some situations not have sufficient time between cleaning events to fully recover from gill damage.

### Pre-exposure to hydroids had no effect on *P*. *perurans* infection rates or AGD development

The average AGD gill scores of the PP- and H+PP-groups were 2.2 at two weeks and 3.9 at five weeks following exposure to *P*. *perurans*. These values are comparable to the characteristic development of amoebic gill disease in the absence of treatment [33, 34]. Despite the pathological changes in the gills caused by hydroid exposure, neither the *P*. *perurans* infection rate nor the disease progression of AGD was noticeably affected. We offer three possible explanations or conclusions from these results:

(1) *Gill damage from exposure to hydroids had no effect on AGD in Atlantic salmon*. Both synergistic and antagonistic interactions between concomitant occurring pathogens are known for Atlantic salmon [45]. Lhorente et al. [46] presumed that skin lesions caused by sea lice *Caligus rogercresseyi* facilitated the infection with *Piscirickettsia salmonis*, which resulted in increased mortality of Atlantic salmon. It has been suspected for some time that pre-existing gill lesions might affect infections with *P*. *perurans* in salmonids [47–49]. An experimental assessment conducted by Adams et al. [50] and involving mechanical injury of Atlantic salmon gills by scalpel or swab treatments, found no effect on AGD severity or infection rates. It must be recognised, however, that trauma-induced damage such as that examined by Adams et al [50], is not the same as gill damage caused by nematocysts and envenomation from hydroids. These studies therefore cannot be directly compared.

(2) *The intensity of the hydroid-inflicted damage on the gills was below the threshold to affect the development of AGD*. Although there were more thrombi found in fish exposed to hydroids than in the control fish for up to 7 days after exposure, the impact on the macroscopic non-specific gill score was limited to the first 24 hrs after exposure. While this difference was statistically significant it may not have been biologically relevant. This experiment simulated a single cleaning event and the associated one-off exposure of salmon to hydroid material. During the main biofouling season in the summer months, when hydroid abundance peaks [51], fish in many Norwegian salmon farms are subjected to net cleaning as frequent as once a week [16]. Hydroid recruitment rates within farms can be immense [20, 23] and if the cleaning process is not fully effective - a frequently occurring phenomenon– damaged hydroids can regenerate within only 5 days [23]. On large farms, net cleaning is a continuing process leading to a *constant* exposure of the fish to low concentrations of suspended hydroid material from neighbouring cages, or ~ weekly exposure to hydroid fragments from repeat cleaning events of ‘their’ cage. Our experiments did not simulate repeated or long-term exposure to hydroid material and thus did not create scenarios where fish lack the time for recovery between insults. While repeat-exposure experiments in controlled conditions are logistically challenging, they are necessary to determine the effect of net cleaning on gill health in realistic scenarios.

The concentration of cleaning waste applied in this experiment was calculated based on hydroid growth patterns and cleaning waste concentrations measured at a restricted number of Norwegian salmon farms. While we are confident in the relevance of these concentrations we are likely to not have simulated ‘extreme’ conditions that may occur on some farms. The experimental concentrations applied by Baxter et al. [24] resulted in a 40-times higher concentration of hydroid fragments compared to this experiment. We suspect that Baxter et al.’s [24] concentrations were substantially higher than what occurs in most farms, but this difference does highlight the paucity of field data available to guide experimental conditions.

(3) *The hydroid-related gill damage and its impact on AGD development were masked by the background pathology present in the fish*. The naïve fish used in this experiment were found to be infected with *B*. *cysticola* and had an average non-specific gill score of 0.4 ± 0.13 and a hyperplasia score of 1.4 ± 0.20. These figures indicate that gills in the naïve fish were not 100% healthy prior to exposure to hydroids or amoebae. This pre-existing gill pathology may have masked effects of hydroids and/or amoebae and may have increased the variability in the data, obscuring differences between treatment groups. The high prevalence of proliferation in the gill epithelium may furthermore have reduced the surface of the gills and the area available for contact with hydroid particles. Paradoxically, this may have protected the gills from more damage by hydroids. Adams et al. [50] observed a lack of attachment of *P*. *perurans* to mechanically damaged gill areas. The possible explanation that a gill immune response created an unfavourable environment for the amoebae could also be true for gill tissue affected by cnidarian barbs and toxins.

### AGD in the hydroid-only treatment group?

From 21 dphe onwards, fish with positive AGD scores were found in tanks that had received hydroid material but not amoebae (H-group). The presence of *P*. *perurans* was confirmed through qPCR for samples from 28 and 35 dphe. No fish with confirmed AGD-infection were encountered in control tanks without hydroids (C-group). Although 80% of the fish in the H-group had positive AGD scores by the end of the experiment, gill scores were significantly lower compared to the treatment group that had been exposed to *P*. *perurans* (PP-group), and AGD lesions or amoeba were not found in the samples. This indicated a delayed development of the disease in the H-group compared to the designated *P*. *perurans* treatment groups [52].

We suspect that the reason for the presence of positive AGD scores in the hydroids-only treatment group is that *P*. *perurans* was accidentally introduced with the hydroid material collected from the field. The hydroids were collected from a farm that, shortly after the retrieval of the samples, reported positive qPCR results for *P*. *perurans* during routine monitoring, showing that, although the fish on site did not express clinical AGD signs, the amoeba was present in the population. Hydroids sampled at farms with AGD outbreaks have been tested positive for *P*. *perurans* and are suspected to act as potential reservoirs for the amoeba [7, 53]. Thus, the hydroid material used in the experiment may have been contaminated with *P*. *perurans*. A comparatively low number of amoebae attached to the hydroid material could explain the slow development of the disease in those tanks [32, 54].

Unfortunately, it was not possible in retrospect to prove this theory. The possibility of an introduction of AGD with material from the field does, however, underscore the need for more research into the reservoirs for *P*. *perurans* and the risk of spreading this disease for example through net cleaning activities [7, 16, 53].

### Multifactorial infections

The infection of the fish with *Ca*. B. cysticola may part been responsible for the underlying inflammatory and degenerative changes that could be seen in fish from all treatment groups. Infections with this pathogen, as well as the SGPV, are common in Norwegian salmon culture [3, 55] and thus may have contributed to a more realistic challenge situation in this experiment. While the fish were likely already infected with *Ca*. B. cysticola when coming from the hatchery, the origin of the SGPV infection cannot be resolved in retrospect.

### Implications for salmon aquaculture

The results of this study convey the important message for global salmon farmers—that even short-term and single exposure to hydroids can affect gill health. This study used conservative calculations to determine a realistic on-farm concentration; however, it is conceivable that actual *in-situ* concentrations and resulting health and welfare effects on fish in many locations may surpass those observed here. Furthermore, even though gill damage due to hydroids may not facilitate AGD, potential interactions with other pathogens that target the gills (but were not included in this study) are possible and need to be examined further before they can be ruled out. Finally, the reverse case, where fish with gills damaged by *P*. *perurans* are being further exposed to cleaning waste containing cnidarians, should be considered. Both stressor sequences are likely to occur in the present-day farming operations and may result in different gill damage.

### Conclusions and future research needs

One major challenge associated with the increase in aquaculture production is the development and implementation of effective disease prevention and treatment [56–58]. This includes a better understanding of the possible interactions between histological gill lesions in farmed salmon and exposure to cnidarian biofouling organisms; thus understanding the role of cnidarian biofouling in multifactorial gill diseases [2, 7, 24]. This study confirmed the negative impact hydroids can have on salmon gill health and highlighted the potential risks that net cleaning pose to fish welfare. However, *in-situ* measurements of gill health before and after net cleaning conducted in the field are necessary to validate these findings. Both laboratory and field research should examine situations where salmon are subject to repeated exposure to cnidarian cleaning waste at realistic intervals. Due to the complexity of disease agents potentially present on salmon farms at any one time, it is necessary to examine hydroids in the context of multifactorial diseases and as potential reservoirs and subsequent carriers of disease agents.

## Supporting information

S1 Appendix Estimation of *Ectopleura larynx* concentration in a salmon cage during net cleaning. (PDF)

S2 Appendix Detailed statistical results. (PDF)

S1 Table Original data. (XLSX)
