# Supplementary material for: Effects of cnidarian biofouling on salmon gill health and development of amoebic gill disease
Source: PLoS One. 2018 Jul 6;13(7):e0199842. doi: 10.1371/journal.pone.0199842 (PMC6034824; doi:10.1371/journal.pone.0199842)
Supplement: S1 Appendix — (PDF) [file pone.0199842.s001.pdf]

Estimation of *Ectopleura larynx* concentration in a salmon cage during net cleaning

The calculations were conducted for a commercial salmon cage with a conical net of the following dimensions: 50 m diameter and 25 m total depth, with a bottom ring in 15 m depth, creating a straight vertical sidewall of 15 m, followed by a 10 m conical tip.

Relevant surface area of the net

It is a common strategy to first clean the vertical sides of a net cage before continuing with the conical bottom. Hydroid material (polyps, fragments, larvae) released into the water are negatively buoyant [1], resulting in the cleaning waste being suspended for a limited time. Because of this sinking behaviour and the time it takes to clean an entire cage, in our calculations of the concentration of cleaning waste in the cage we considered only the upper 15 m of the net. This portion of the cage comprises a **surface area of 2 356 m^2** which was defined as the biofouled net area supplying cleaning waste.

Impacted water volume

The distribution of cleaning waste through the volume of a cage is not fully understood. Based on visual observations and on-going research into the dispersal of cleaning waste, the calculations were based on the assumption that cleaning waste is largely restricted to a horizontal distance of 10 m from the net wall (S1 Fig 1). Based on this assumption, a **total impact volume of 18 850 m^3** was derived.

Hydroid density on the net and in the water

The reported density of hydroid polyps growing on salmon cage nets ranges from 40 000 polyps m^{-2} [2] to 112 000 polyps m^{-2} ([3] – based on a hydroid density of 1.4 polyps mm^{-1} net twine, scaled to a 25 mm half-mesh net). Given the assumptions described above, these densities of hydroids on cage nets would result in a concentration of 5 000 to 14 000 polyps m^{-3}.

Given the cleaning process not always removes all biofouling from the net, a **concentration of 10 000 polyps m^{-3}** was chosen for the experiment to represent conditions representative of *in situ* net cleaning operations.

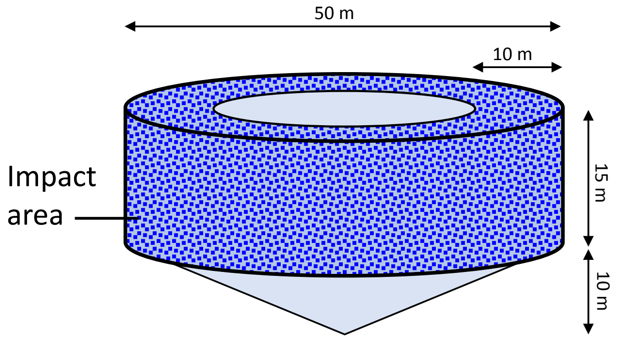

S1 Fig 1: Schematic overview of a salmon cage. The shaded zone indicates the area impacted by cleaning waste.

References

1. Mackie GO. Growth of the hydroid *Tubularia* in culture. Symp Zool Soc London. 1966;16:397-412.
2. Baxter EJ, Sturt MM, Ruane NM, Doyle TK, McAllen R, Rodger HD. Biofouling of the hydroid *Ectopleura larynx* on aquaculture nets in Ireland: implications for finfish health. Fish Vet J. 2012;13:17-29.
3. Lader P, Fredriksson D, Guenther J, Volent Z, Bloecher N, Kristiansen D, et al. Drag on hydroid-fouled nets — An experimental approach. China Ocean Eng. 2015;29(3):369-89.
