# Supplementary material for: Effects of cnidarian biofouling on salmon gill health and development of amoebic gill disease
Source: PLoS One. 2018 Jul 6;13(7):e0199842. doi: 10.1371/journal.pone.0199842 (PMC6034824; doi:10.1371/journal.pone.0199842)
Supplement: S2 Appendix — (PDF) [file pone.0199842.s002.pdf]

# Detailed statistical results

Significant terms are marked in red

## Non-specific gill score

### AVERAGE Non-specific gill score

**Exposure day + 1 dphe**

*PERMANOVA table of results*

| Source | df | SS | MS | Pseudo-F | P(perm) |
|---|---|---|---|---|---|
| Treatment | 1 | 1.5313 | 1.5313 | 5.8136 | 0.0245 |
| Time | 1 | 0.28125 | 0.28125 | 1.0678 | 0.3216 |
| Treatment x Time | 1 | 0.78125 | 0.78125 | 2.9661 | 0.098 |
| Residuals | 28 | 7.375 | 0.26339 | | |
| Total | 31 | 9.9688 | | | |

**3 dphe onwards**

*PERMANOVA table of results*

| Source | df | SS | MS | Pseudo-F | P(perm) | P(MC) |
|---|---|---|---|---|---|---|
| Treatment | 3 | 0.083333 | 0.027778 | 0.13333 | | 0.9409 |
| Time | 5 | 1.65 | 0.33 | 1.1061 | 0.3912 | |
| Tank (Treatment) | 4 | 0.83333 | 0.20833 | 0.90909 | 0.4734 | |
| Treatment x Time | 15 | 5.3167 | 0.35444 | 1.1881 | 0.3498 | |
| Tank (Treatment) x Time | 20 | 5.9667 | 0.29833 | 1.3018 | 0.1726 | |
| Residuals | 192 | 44 | 0.22917 | | | |
| Total | 239 | 57.85 | | | | |

*Pooled terms*
Tank (Treatment) x Time

*PERMANOVA table of results*

| Source | df | SS | MS | Pseudo-F | P(perm) | P(MC) |
|---|---|---|---|---|---|---|
| Treatment | 3 | 0.083333 | 0.027778 | 0.13333 | | 0.9382 |
| Time | 5 | 1.65 | 0.33 | 1.4001 | 0.2168 | |
| Tank (Treatment) | 4 | 0.83333 | 0.20833 | 0.88392 | 0.4814 | |
| Treatment x Time | 15 | 5.3167 | 0.35444 | 1.5038 | 0.1068 | |
| Pooled | 212 | 49.967 | 0.23569 | | | |
| Total | 239 | 57.85 | | | | |

*Pooled terms*
Tank (Treatment)
Tank (Treatment) x Time

*PERMANOVA table of results*

| Source | df | SS | MS | Pseudo-F | P(perm) |
|---|---|---|---|---|---|
| Treatment | 3 | 0.083333 | 0.027778 | 0.11811 | 0.949 |
| Time | 5 | 1.65 | 0.33 | 1.4031 | 0.2176 |
| Treatment x Time | 15 | 5.3167 | 0.35444 | 1.5071 | 0.0961 |
| Pooled | 216 | 50.8 | 0.23519 | | |
| Total | 239 | 57.85 | | | |

No significant differences

## PREVALENCE for positive non-specific gill score

*Fishers Exact Test:*

| C-group vs. H-group | p |
|---|---|
| Exposure day | 0.343 |
| 1 dphe | 0.029 |

# AGD gill score

## Average AGD score

### 3 dphe onwards

*PERMANOVA table of results*

| Source | df | SS | MS | Pseudo-F | P(perm) | P(MC) |
|---|---|---|---|---|---|---|
| Treatment | 3 | 218.02 | 72.672 | 792.79 | | 0.0001 |
| Time | 5 | 97.233 | 19.447 | 60.456 | 0.0001 | |
| Tank (Treatment) | 4 | 0.36667 | 0.091667 | 0.47826 | 0.7633 | |
| Treatment x Time | 15 | 68.733 | 4.5822 | 14.245 | 0.0001 | |
| Tank (Treatment) x Time | 20 | 6.4333 | 0.32167 | 1.6783 | 0.0398 | |
| Residuals | 192 | 36.8 | 0.19167 | | | |
| Total | 239 | 427.58 | | | | |

*PAIR-WISE TESTS*

Term 'Treatment x Time'

Within level '3 dphe' of factor 'time'

| Groups | t | P(MC) |
|---|---|---|
| Control, hydroids | Denominator is 0 | |
| Control, P.per. | 6 | 0.0001 |
| Control, Hydroids + P.per. | 3 | 0.0076 |
| Hydroids, P.per. | 6 | 0.0001 |
| Hydroids, Hydroids + P.per. | 3 | 0.0079 |
| P.per., Hydro + P.per. | 1.4056 | 0.1722 |

Within level '7 dphe' of factor 'time'

| Groups | t | P(MC) |
|---|---|---|
| Control, hydroids | Denominator is 0 | |
| Control, P.per. | 1.964 | 0.0668 |
| Control, Hydroids + P.per. | 2.3333 | 0.0303 |
| Hydroids, P.per. | 1.964 | 0.0672 |
| Hydroids, Hydroids + P.per. | 2.3333 | 0.0338 |
| P.per., Hydro + P.per. | 1.1882 | 0.2503 |

Within level '14 dphe' of factor 'time'

| Groups | t | P(MC) |
|---|---|---|
| Control, hydroids | Denominator is 0 | |
| Control, P.per. | 9 | 0.0001 |
| Control, Hydroids + P.per. | Denominator is 0 | |
| Hydroids, P.per. | 9 | 0.0001 |
| Hydroids, Hydroids + P.per. | Denominator is 0 | |
| P.per., Hydroids + P.per. | 1.5 | 0.1539 |

Within level '21 dphe' of factor 'time'

| Groups | t | P(MC) |
|---|---|---|
| Control, hydroids | 1 | 0.3212 |
| Control, P.per. | 15 | 0.0001 |
| Control, Hydroids + P.per. | 16.5 | 0.0001 |
| Hydroids, P.per. | 12.348 | 0.0001 |
| Hydroids, Hydroids + P.per. | 12.6 | 0.0001 |
| P.per., Hydroids + P.per. | 1.4056 | 0.1764 |

Within level '28 dphe' of factor 'time'

| Groups | t | P(MC) |
|---|---|---|
| Control, hydroids | 1 | 0.3308 |
| Control, P.per. | 15.922 | 0.0001 |
| Control, Hydroids + P.per. | 11 | 0.0001 |
| Hydroids, P.per. | 13.056 | 0.0001 |
| Hydroids, Hydroids + P.per. | 9.3915 | 0.0001 |
| P.per., Hydroids + P.per. | 1.5492 | 0.1386 |

Within level '35 dphe' of factor 'time'

| Groups | t | P(MC) |
|---|---|---|
| Control, hydroids | 5.0138 | 0.0001 |
| Control, P.per. | 17.335 | 0.0001 |
| Control, Hydroids + P.per. | 18.974 | 0.0001 |
| Hydroids, P.per. | 10.04 | 0.0001 |
| Hydroids, Hydroids + P.per. | 11.196 | 0.0001 |
| P.per., Hydroids + P.per. | 1 | 0.3332 |

# Thrombi score

### Exposure day + 1 dphe

*PERMANOVA table of results*

| Source | df | SS | MS | Pseudo-F | P(perm) |
|---|---|---|---|---|---|
| Treatment | 1 | 7.0313 | 7.0313 | 28.636 | 0.0001 |
| Time | 1 | 9.0313 | 9.0313 | 36.782 | 0.0001 |
| Treatment x Time | 1 | 7.0313 | 7.0313 | 28.636 | 0.0001 |
| Residuals | 28 | 6.875 | 0.24554 | | |
| Total | 31 | 29.969 | | | |

*PAIR-WISE TESTS*

Term 'Treatment x Time' for pairs of levels of factor 'Treatment'

Within level 'Exposure day' of factor 'Time'

| Groups | t | P(perm) |
|---|---|---|
| Control. hydroids | Denominator is 0 | |

Within level '1 dphe' of factor 'Time'

| Groups | t | P(MC) |
|---|---|---|
| Control. hydroids | 5.3513 | 0.0001 |

### 3 dphe onwards

*PERMANOVA table of results*

| Source | df | SS | MS | Pseudo-F | P(perm) | P(MC) |
|---|---|---|---|---|---|---|
| Treatment | 3 | 12.713 | 4.2375 | 19.189 | | 0,0092 |
| Time | 5 | 21.387 | 4.2775 | 15.794 | 0.0002 | |
| Tank (Treatment) | 4 | 0.88333 | 0.22083 | 0.99065 | 0.4057 | |
| Treatment x Time | 15 | 22.363 | 1.4908 | 5.5046 | 0.0003 | |
| Tank (Treatment) x Time | 20 | 5.4167 | 0.27083 | 1.215 | 0.2534 | |
| Residuals | 192 | 42.8 | 0.22292 | | | |
| Total | 239 | 105.56 | | | | |

*Pooled terms*
Tank (Treatment) x Time

*PERMANOVA table of results*

| Source | df | SS | MS | Pseudo-F | P(perm) | P(MC) |
|---|---|---|---|---|---|---|
| Treatment | 3 | 12.713 | 4.2375 | 19.189 | | 0,0072 |
| Time | 5 | 21.387 | 4.2775 | 18.807 | 0.0001 | |
| Tank (Treatment) | 4 | 0.88333 | 0.22083 | 0.97096 | 0.424 | |
| Treatment x Time | 15 | 22.363 | 1.4908 | 6.5549 | 0.0001 | |
| Pooled | 212 | 48.217 | 0.22744 | | | |
| Total | 239 | 105.56 | | | | |

*Pooled terms*
Tank (Treatment)
Tank (Treatment) x Time

*PERMANOVA table of results*

| Source | df | SS | MS | Pseudo-F | P(perm) |
|---|---|---|---|---|---|
| Treatment | 3 | 12.713 | 4.2375 | 18.642 | 0.0001 |
| Time | 5 | 21.387 | 4.2775 | 18.818 | 0.0001 |
| Treatment x Time | 15 | 22.363 | 1.4908 | 6.5585 | 0.0001 |
| Pooled | 216 | 49.1 | 0.22731 | | |
| Total | 239 | 105.56 | | | |

*PAIR-WISE TESTS*

Term 'Treatment x Time' for pairs of levels of factor 'Treatment'

Within level '3 dphe' of factor 'Time'

| Groups | t | P(MC) |
|---|---|---|
| Control, Hydroids | 6.862 | 0.0001 |
| Control, P.per | 1.5 | 0.1493 |
| Control, Hydroids + P.per | 5.5816 | 0.0001 |
| Hydroids, P.per | 5.5317 | 0.0001 |
| Hydroids, Hydroids + P.per | 1.0366 | 0.3094 |
| P.Per, Hydroids + P.per | 4.3333 | 0.0004 |

Within level '7 dphe' of factor 'Time'

| Groups | t | P(MC) |
|---|---|---|
| Control, Hydroids | 2.2778 | 0.0365 |
| Control, P.per | Negative | |
| Control, Hydroids + P.per | 3.8571 | 0.0011 |
| Hydroids, P.per | 2.2778 | 0.0334 |
| Hydroids, Hydroids + P.per | 0.55709 | 0.5883 |
| P.Per, Hydroids + P.per | 3.8571 | 0.0011 |

Within level '14 dphe' of factor 'Time'

| Groups | t | P(MC) |
|---|---|---|
| Control, Hydroids | Denominator is 0 | |
| Control, P.per | 1 | 0.3358 |
| Control, Hydroids + P.per | 1.4056 | 0.1802 |
| Hydroids, P.per | 1 | 0.328 |
| Hydroids, Hydroids + P.per | 1.4056 | 0.1766 |
| P.Per, Hydroids + P.per | 0.84853 | 0.4053 |

Within level '21 dphe' of factor 'Time'

| Groups | t | P(MC) |
|---|---|---|
| Control, Hydroids | Negative | |
| Control, P.per | 1 | 0.3272 |
| Control, Hydroids + P.per | 1.2362 | 0.2297 |
| Hydroids, P.per | 1 | 0.3375 |
| Hydroids, Hydroids + P.per | 1.2362 | 0.2338 |
| P.Per, Hydroids + P.per | 1.8091 | 0.089 |

Within level '28 dphe' of factor 'Time'

| Groups | t | P(MC) |
|---|---|---|
| Control, Hydroids | 0.84853 | 0.3997 |
| Control, P.per | Negative | |
| Control, Hydroids + P.per | 1.44E-08 | 1 |
| Hydroids, P.per | 0.84853 | 0.3991 |
| Hydroids, Hydroids + P.per | 0.84853 | 0.4084 |
| P.Per, Hydroids + P.per | Negative | |

Within level '35 dphe' of factor 'Time'

| Groups | t | P(MC) |
|---|---|---|
| Control, Hydroids | 1 | 0.3215 |
| Control, P.per | Negative | |
| Control, Hydroids + P.per | Negative | |
| Hydroids, P.per | 1 | 0.3387 |
| Hydroids, Hydroids + P.per | 1 | 0.3295 |
| P.Per, Hydroids + P.per | Negative | |

## PREVALENCE for positive Thrombi score

*Fishers Exact Test:*

| C-group vs. H-group | p |
|---|---|
| Exposure day | 0.342 |
| 1 dphe | 0.01 |

# Hyperplasia score

## AVERAGE Hyperplasia score

**Exposure day + 1 dphe**

*PERMANOVA table of results*

| Source | df | SS | MS | Pseudo-F | P(perm) |
|---|---|---|---|---|---|
| Treatment | 1 | 1.5313 | 1.5313 | 3.0901 | 0.0946 |
| Time | 1 | 0.03125 | 0.03125 | 0.063063 | 0.8315 |
| Treatment x Time | 1 | 1.5313 | 1.5313 | 3.0901 | 0.0906 |
| Residuals | 28 | 13.875 | 0.49554 | | |
| Total | 31 | 16.969 | | | |

No significant differences

**3 dphe onwards**

*PERMANOVA table of results*

| Source | df | SS | MS | Pseudo-F | P(perm) | P(MC) |
|---|---|---|---|---|---|---|
| Treatment | 3 | 14.733 | 4.9111 | 147.33 | | 0,0002 |
| Time | 5 | 24.583 | 4.9167 | 8.1492 | 0.0006 | |
| Tank (Treatment) | 4 | 0.13333 | 0.033333 | 0.048193 | 0.9961 | |
| Treatment x Time | 15 | 29.617 | 1.9744 | 3.2726 | 0.0089 | |
| Tank (Treatment) x Time | 20 | 12.067 | 0.60333 | 0.87229 | 0.6216 | |
| Residuals | 192 | 132.8 | 0.69167 | | | |
| Total | 239 | 213.93 | | | | |

*Pooled terms*
Tank (Treatment) x Time

*PERMANOVA table of results*

| Source | df | SS | MS | Pseudo-F | P(perm) | P(MC) |
|---|---|---|---|---|---|---|
| Treatment | 3 | 14.733 | 4.9111 | 147.33 | | 0,0002 |
| Time | 5 | 24.583 | 4.9167 | 7.1951 | 0.0001 | |
| Tank (Treatment) | 4 | 0.13333 | 0.033333 | 0.04878 | 0.995 | |
| Treatment x Time | 15 | 29.617 | 1.9744 | 2.8894 | 0.0006 | |
| Residuals | 212 | 144.87 | 0.68333 | | | |
| Total | 239 | 213.93 | | | | |

*Pooled terms*
Tank (Treatment)
Tank (Treatment) x Time

*PERMANOVA table of results*

| Source | df | SS | MS | Pseudo-F | P(perm) |
|---|---|---|---|---|---|
| Treatment | 3 | 14.733 | 4.9111 | 7.3159 | 0.0002 |
| Time | 5 | 24.583 | 4.9167 | 7.3241 | 0.0001 |
| Treatment x Time | 15 | 29.617 | 1.9744 | 2.9412 | 0.0004 |
| Pooled | 216 | 145 | 0.6713 | | |
| Total | 239 | 213.93 | | | |

*PAIR-WISE TESTS*

Term 'Treatment x Time' for pairs of levels of factor

Within level '3 dphe' of factor 'Time'

| Groups | t | P(MC) |
|---|---|---|
| Control, Hydroids | 0.67082 | 0.523 |
| Control, P.per | 3.82E-08 | 1 |
| Control, Hydroids + P.per | 1.3416 | 0.1978 |
| Hydroids, P.per | 0.47434 | 0.6312 |
| Hydroids, Hydroids + P.per | 0.6396 | 0.5316 |
| P.Per, Hydroids + P.per | 0.94868 | 0.3548 |

Within level '7 dphe' of factor 'Time'

| Groups | t | P(MC) |
|---|---|---|
| Control, Hydroids | 0.25631 | 0.8027 |
| Control, P.per | 1.47E-08 | 1 |
| Control, Hydroids + P.per | 0.70065 | 0.489 |
| Hydroids, P.per | 0.3046 | 0.7621 |
| Hydroids, Hydroids + P.per | 1.1442 | 0.2634 |
| P.Per, Hydroids + P.per | 0.80498 | 0.4275 |

Within level '14 dphe' of factor 'Time'

| Groups | t | P(MC) |
|---|---|---|
| Control, Hydroids | 0.46291 | 0.6541 |
| Control, P.per | 0.43759 | 0.6688 |
| Control, Hydroids + P.per | 1.5213 | 0.1473 |
| Hydroids, P.per | 1.78E-08 | 1 |
| Hydroids, Hydroids + P.per | 2.4 | 0.0262 |
| P.Per, Hydroids + P.per | 2.1909 | 0.0365 |

Within level '21 dphe' of factor 'Time'

| Groups | t | P(MC) |
|---|---|---|
| Control, Hydroids | 0.91499 | 0.3726 |
| Control, P.per | 1.6348 | 0.1214 |
| Control, Hydroids + P.per | 0.3873 | 0.7035 |
| Hydroids, P.per | 4.0931 | 0.0009 |
| Hydroids, Hydroids + P.per | 1.5213 | 0.1438 |
| P.Per, Hydroids + P.per | 1.3007 | 0.2061 |

Within level '28 dphe' of factor 'Time'

| Groups | t | P(MC) |
|---|---|---|
| Control, Hydroids | 1.89E-08 | 1 |
| Control, P.per | 2.4495 | 0.0247 |
| Control, Hydroids + P.per | 2.3333 | 0.0319 |
| Hydroids, P.per | 2.2283 | 0.0411 |
| Hydroids, Hydroids + P.per | 2.0896 | 0.0507 |
| P.Per, Hydroids + P.per | 0.39736 | 0.6942 |

Within level '35 dphe' of factor 'Time'

| Groups | t | P(MC) |
|---|---|---|
| Control, Hydroids | 0.84665 | 0.4079 |
| Control, P.per | 6.3258 | 0.0001 |
| Control, Hydroids + P.per | 6.5485 | 0.0001 |
| Hydroids, P.per | 4.4272 | 0.0006 |
| Hydroids, Hydroids + P.per | 4.6663 | 0.0002 |
| P.Per, Hydroids + P.per | 0.44721 | 0.6585 |

## PREVALENCE for positive Hyperplasia score

*Fishers Exact Test:*

| C-group vs. H-group | p |
|---|---|
| Exposure day | 0.342 |
| 1 dphe | 0.01 |

# AGD lesion score

## AVERAGE AGD lesion score

**3 dphe onwards**

*PERMANOVA table of results*

| Source | df | SS | MS | Pseudo-F | P(perm) | P(MC) |
|---|---|---|---|---|---|---|
| Treatment | 3 | 27.546 | 9.1819 | 48.97 | | 0.0013 |
| Time | 5 | 39.237 | 7.8475 | 101.26 | 0.0001 | |
| Tank (Treatment) | 4 | 0.75 | 0.1875 | 1.9565 | 0.101 | |
| Treatment x Time | 15 | 40.179 | 2.6786 | 34.563 | 0.0001 | |
| Tank (Treatment) x Time | 20 | 1.55 | 0.0775 | 0.8087 | 0.709 | |
| Residuals | 192 | 18.4 | 0.095833 | | | |
| Total | 239 | 127.66 | | | | |

*Pooled terms*

Tank (Treatment) x Time

*PERMANOVA table of results*

| Source | df | SS | MS | Pseudo-F | P(perm) | P(MC) |
|---|---|---|---|---|---|---|
| Treatment | 3 | 27.546 | 9.1819 | 48.97 | | 0.002 |
| Time | 5 | 39.237 | 7.8475 | 83.392 | 0.0001 | |
| Tank (Treatment) | 4 | 0.75 | 0.1875 | 1.9925 | 0.0979 | |
| Treatment x Time | 15 | 40.179 | 2.6786 | 28.464 | 0.0001 | |
| Residuals | 212 | 19.95 | 0.094104 | | | |
| Total | 239 | 127.66 | | | | |

*PAIR-WISE TESTS*

Term 'Treatment x Time' for pairs of levels of factor 'Treatment'

Too little data available for 3, 7 and 14 dphe. Treatment groups "Control" and "Hydroids" had only 0 values

Within level '21 dphe' of factor 'Time'

| Groups | t | P(perm) | P(MC) |
|---|---|---|---|
| Control, Hydroids | Denominator is 0 | | |
| Control, P.per | 5 | 0,3368 | 0,0394 |
| Control, Hydroids + P.per | 1 | 1 | 0,427 |
| Hydroids, P.per | 5 | 0,3386 | 0,0379 |
| Hydroids, Hydroids + P.per | 1 | 1 | 0,4192 |
| P.Per, Hydroids + P.per | 1,3416 | 0,6626 | 0,3141 |

Within level '28 dphe' of factor 'Time'

| Groups | t | P(perm) | P(MC) |
|---|---|---|---|
| Control, Hydroids | Denominator is 0 | | |
| Control, P.per | Denominator is 0 | | |
| Control, Hydroids + P.per | 4,3333 | 0,3234 | 0,0553 |
| Hydroids, P.per | Denominator is 0 | | |
| Hydroids, Hydroids + P.per | 4,3333 | 0,3358 | 0,0502 |
| P.Per, Hydroids + P.per | 1 | 1 | 0,4308 |

Within level '35 dphe' of factor 'Time'

| Groups | t | P(perm) | P(MC) |
|---|---|---|---|
| Control, Hydroids | Denominator is 0 | | |
| Control, P.per | Denominator is 0 | | |
| Control, Hydroids + P.per | 11 | 0,3277 | 0,0092 |
| Hydroids, P.per | Denominator is 0 | | |
| Hydroids, Hydroids + P.per | 11 | 0,3245 | 0,0076 |
| P.Per, Hydroids + P.per | 1 | 1 | 0,4276 |

## PREVALENCE for positive AGD lesion score

*Fishers Exact Test*:*

| PP- vs. H+PP group | p |
|---|---|
| 21 dphe | 0.626 |
| 28 dphe | 0.1 |

*Conducted for days with sufficient data
